# Decreased amygdala volume in adults after premature birth

**DOI:** 10.1038/s41598-021-84906-2

**Published:** 2021-03-08

**Authors:** Benita Schmitz-Koep, Juliana Zimmermann, Aurore Menegaux, Rachel Nuttall, Josef G. Bäuml, Sebastian C. Schneider, Marcel Daamen, Henning Boecker, Claus Zimmer, Dieter Wolke, Peter Bartmann, Dennis M. Hedderich, Christian Sorg

**Affiliations:** 1 Department of Diagnostic and Interventional Neuroradiology, School of Medicine, Technical University of Munich, Klinikum Rechts Der Isar, Ismaninger Str. 22, 81675 Munich, Germany; 2 TUM-NIC Neuroimaging Center, School of Medicine, Technical University of Munich, Ismaninger Str. 22, 81675 Munich, Germany; 3 Functional Neuroimaging Group, Department of Diagnostic and Interventional Radiology, University Hospital Bonn, Venusberg-Campus 1, Bonn, Germany; 4 Department of Neonatology, University Hospital Bonn, Venusberg-Campus 1, Bonn, Germany; 5 Department of Psychology, University of Warwick, University Road, Coventry, CV4 7AL UK; 6 Warwick Medical School, University of Warwick, University Road, Coventry, CV4 7AL UK; 7 Department of Psychiatry, School of Medicine, Technical University of Munich, Ismaninger Str. 22, 81675 Munich, Germany

**Keywords:** Development of the nervous system, Social behaviour

## Abstract

Premature-born infants have impaired amygdala structure, presumably due to increased stress levels of premature birth mediated by the amygdala. However, accounting for lifelong plasticity of amygdala, it is unclear whether such structural changes persist into adulthood. To address this problem, we stated the following questions: first, are whole amygdala volumes reduced in premature-born adults? And second, as adult anxiety traits are often increased after prematurity and linked with amygdala structure, are alterations in amygdala associated with adults’ anxiety traits after premature birth? We addressed these questions by automated amygdala segmentation of MRI volumes in 101 very premature-born adults (< 32 weeks of gestation and/or birth weight below 1500 g) and 108 full-term controls at 26 years of age of a prospectively and longitudinally collected cohort. We found significantly lower whole amygdala volumes in premature-born adults. While premature-born adults had significantly higher T score for avoidant personality reflecting increased social anxiety trait, this trait was not correlated with amygdala volume alterations. Results demonstrate reduced amygdala volumes in premature born adults. Data suggest lasting effects of prematurity on amygdala structure.

## Introduction

Premature birth, i.e. birth before 37 weeks of gestation, is frequent with an increasing worldwide prevalence of almost 11%^1^. It is associated with an increased risk for impaired neurocognitive development, which increases with earlier gestational age (GA)^2–4^. For example, very premature-born adults, i.e. born very preterm (VP) before 32 weeks of GA and/or with very low birth weight (VLBW) below 1500 g, have on average more than 10 points lower full-scale IQ scores compared to full-term (FT) controls, which is mediated by aberrant development of distinct brain structures or processes such as cortical folding, white matter integrity and subcortical grey matter volume^5–23^. Causes of aberrant brain development are inflammatory, hypoxic-ischemic and/or stress-related events, which not only affect vulnerable developmental processes, for example pre-oligodendrocyte or subplate neuron (SPN) development at microscopic level^24–26^, but also lead to altered macroscopic brain development such as cortical gyri development or white matter integrity^7,8,10,12,15,18,27,28^.

Amongst multiple brain systems affected in human prematurity, the amygdala might be interesting for two particular reasons: First, the amygdala mediates the brain’s stress responses, with stress being a critical factor of premature birth; and second, the amygdala mediates anxiety and social anxiety behavior, which are both often increased after premature birth. We will explore these reasons in more detail in the following paragraphs.

Concerning the role of the amygdala in the brain’s stress response in the context of preterm birth, stress is a critical component of prematurity. Especially preterm infants in the neonatal intensive care unit are exposed to many potential stressors, which are associated with altered brain development^29–32^. Examples for these stressors include maternal separation, extra-uterine conditions under conditions of immaturity, pain, diagnostic procedures, and treatment procedures such as mechanical ventilation and potential surgery. More specifically, stress exposure modulates amygdala structure and function, possibly making it vulnerable to prematurity^33–35^. Indeed, reduced whole amygdala volumes have already been described in neonates and in children after premature birth^36,37^. For example, Chau et al.^38^ found that, in VP children, greater exposure to neonatal pain/stress was associated with smaller volume of the amygdala amongst volumes of other structures of the limbic system and basal ganglia. However, accounting for lifelong plasticity of the amygdala^39–41^, it remains unclear whether amygdala volumes are lastingly altered into adulthood after premature birth.

Secondly, it is well-known that the amygdala is functionally associated with anxiety and socially anxious behavior^42–45^. For example, Spampinato et al.^46^ found an inverse correlation between anxiety measures and volume of the amygdala amongst other regions of the limbic system and prefrontal cortex in healthy adults. Remarkably, after premature birth, an increased risk for elevated anxiety traits and anxiety disorders amongst other psychiatric disorders has been reported^2,14^. Furthermore, premature-born individuals have been characterized as less extraverted and more withdrawn^47,48^. For example, recently, Johns et al.^49^ found that social impairments in preterm-born adolescents were associated with functional connectivity of the amygdala, supporting a possible relationship between prematurity, social anxiety and the amygdala. Therefore, we tested the question whether there might be an association between altered amygdala volumes and increased anxiety in premature-born adults.

In summary, we addressed the following questions: First, are whole amygdala volumes altered in premature-born adults? And second, is there an association between alterations in amygdala volumes and anxiety? To answer these questions, we studied 101 very premature-born adults and 108 full-term controls at 26 years of age by the use of structural brain MRI, automated FreeSurfer segmentation of the amygdala in MRI data, and Young Adult Self Report (YASR) assessment to measure anxiety traits.

## Results

### Sample characteristics

Table 1 presents group demographic and clinical background variables. The VP/VLBW group and the FT group did not differ significantly regarding sex (*p* = 0.894) and age at scanning (*p* = 0.165). By design of the study, VP/VLBW subjects had significantly lower GA (*p* < 0.001) and lower birth weight (BW) (*p* < 0.001) compared to FT controls. Furthermore, TIV was significantly smaller in VP/VLBW individuals compared to controls (*p* = 0.001).

**Table 1:** Demographical, clinical and cognitive data.

	VP/VLBW (n = 101)			FT (n = 108)			*p*-value
	Mean	SD	Range	Mean	SD	Range	
Sex (male/female)	58/43			63/45			0.894
Age (years)	26.7	± 0.61	25.7–28.3	26.8	± 0.75	25.5–28.9	0.165
GA (weeks)	30.5	± 2.1	25–36	39.7	± 1.1	37–42	** < 0.001**
BW (g)	1325	± 313	630–2070	3389	± 447	2120–4670	** < 0.001**
Ventilation (days)	12.1	± 17.5	0–81	n.a	n.a	n.a	n.a
TIV (mm^3^)	1457.9	± 141.1	1132.3–1793.6	1524.0	± 146.1	1166.2–1918.7	**0.001**
**YASR DSM-oriented scale, T score**	**Mean**	**SE**	**Range**	**Mean**	**SE**	**Range**	***p***-**value**
Anxiety	52.0	± 4.1	50–70	51.2	± 2.9	50–67	0.097
Avoidant personality	55.5	± 8.0	50–83	52.5	± 4.9	50–73	**0.001**
**Scanner**	**n**	**%**		**n**	**%**		
Bonn Achieva 3T	5	5.0		11	10.2		
Bonn Ingenia 3T	33	32.7		17	15.7		
Munich Achieva 3T	60	59.4		63	58.3		
Munich Ingenia 3T	3	3.0		17	15.7		

Statistical comparisons: sex with χ^2^ statistics; age, GA, BW, TIV and YASR with two sample t-tests. Bold letters indicate statistical significance defined as *p* < 0.05.

BW, birth weight; DSM, Diagnostic and Statistical Manual of Mental Disorders; FT, full-term; GA, gestational age; SD, standard deviation; SE, standard error; VP/VLBW, very preterm and/or very low birthweight, YASR, Young Adult Self Report.

### Reduced amygdala volumes in very premature-born adults

**Figure 1:**
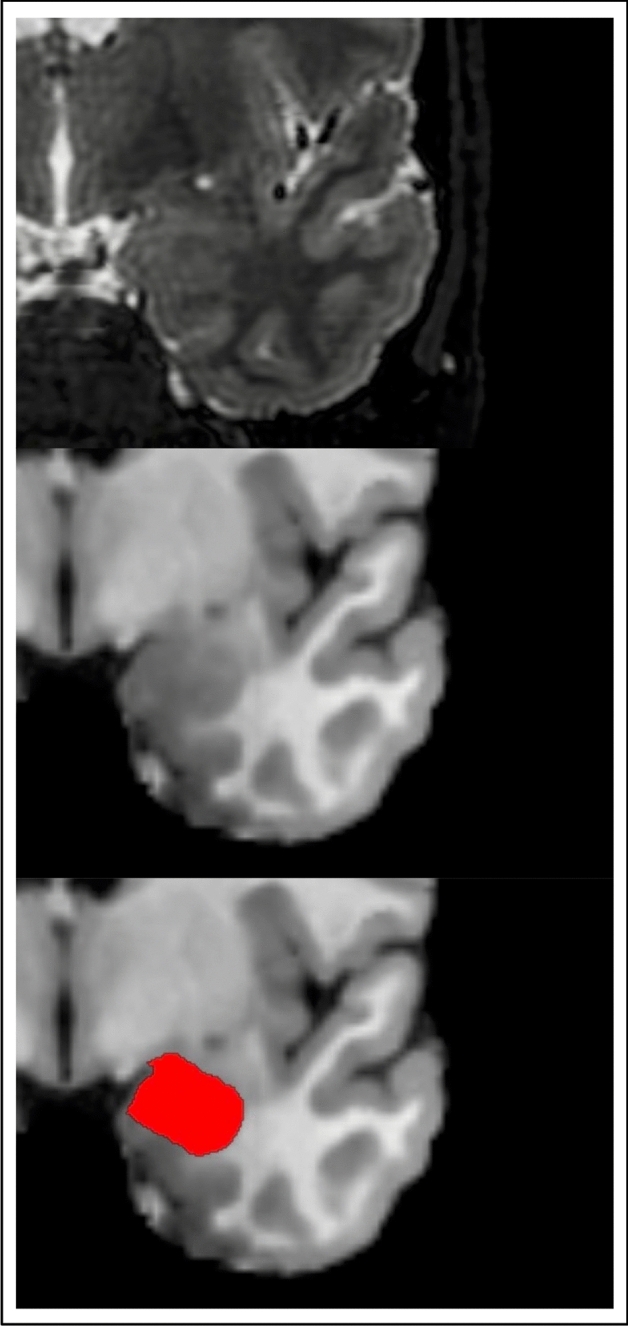
Segmentation of the amygdala. T2-weighted and T1-weighted images of the amygdala as segmented by FreeSurfer.

**Figure 2:**
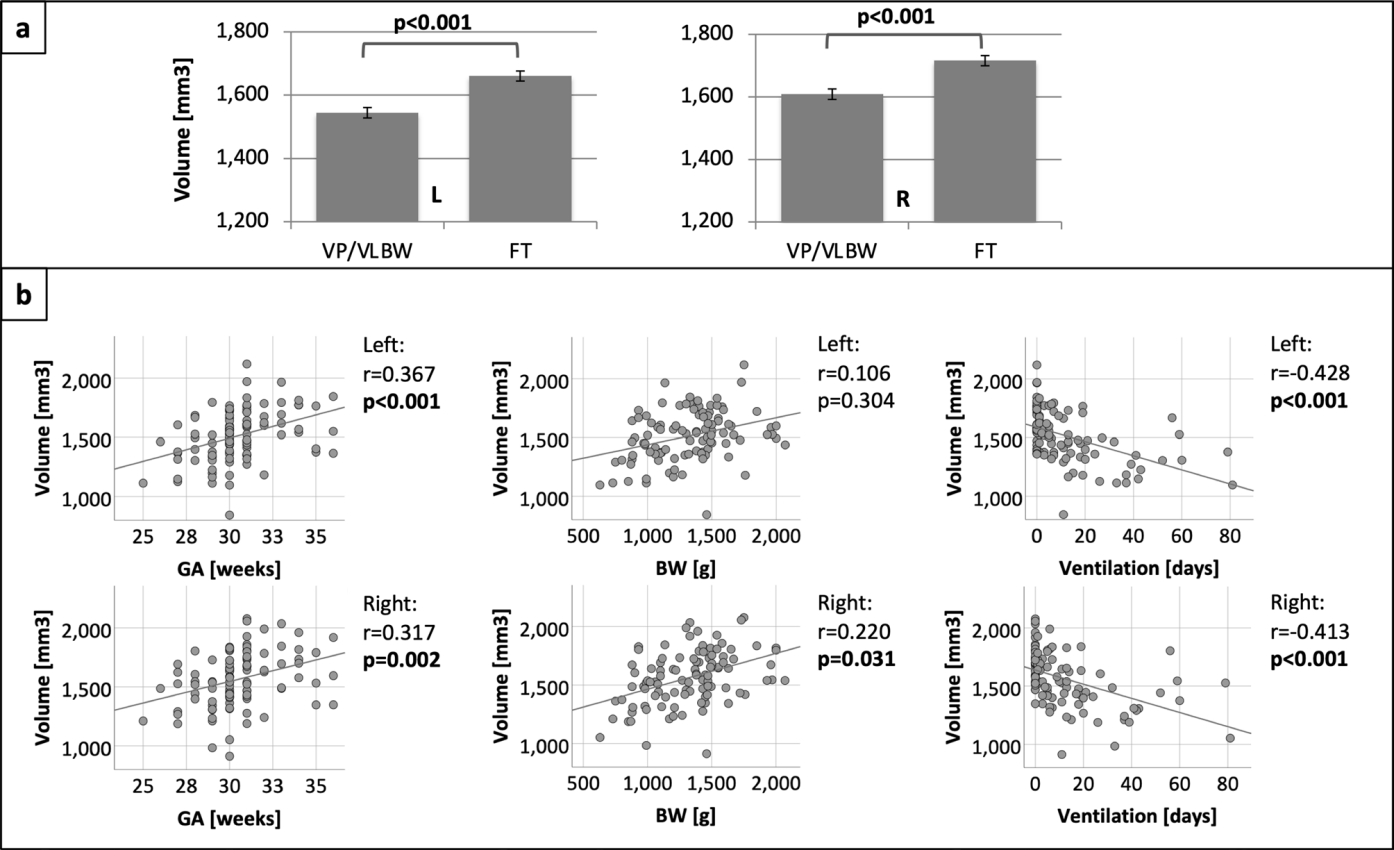
(**A**) Group comparison of whole amygdala volumes. Estimated marginal means of left and right whole amygdala volumes (in mm^3^) are shown as bar charts with SE (in mm^3^) as error bars. Bold letters indicate statistical significance. (**B**) Relationship between whole amygdala volume and variables of premature birth. The associations between left and right whole amygdala volume and GA, BW and duration of ventilation are shown as scatter plots. GA (in weeks), BW (in grams) and duration of ventilation (in days) are plotted on the x-axes. Whole amygdala volumes (in mm^3^) are plotted on the y-axes. Linear regression lines as well as correlation coefficients and *p*-values were added. Bold letters indicate statistical significance after FDR correction using the Benjamini–Hochberg procedure. Abbreviations: BW, birth weight; FT, full term; GA, gestational age; L, left; n. s., not significant; R, right; VP/VLWB, very preterm and/or very low birth weight.

Automated FreeSurfer-based segmentation of the amygdala in structural MRI data is visualized in Fig. 1. First, we tested whether whole amygdala volumes are altered in VP/VLBW adults compared to FT controls using general linear models. Total intracranial volume (TIV), sex and scanner were entered as covariates. We found significantly lower left (*p* < 0.001) and right whole amygdala volumes (*p* < 0.001) in VP/VLBW individuals compared to controls. Figure 2a and Table S1 show estimated marginal means and *p*-values.

In order to control for an impact of intraventricular hemorrhage on our results, we removed these subjects (see Table S3) and repeated general linear model analyses for left and right whole amygdala volumes between the remaining subjects of the VP/VLBW group and the FT group. We found significantly lower left (*p* < 0.001) and right whole amygdala volume (*p* = 0.001) in VP/VLBW subjects without intraventricular hemorrhage compared to FT controls (see Table S4), verifying that amygdala volume is lastingly reduced after premature birth. These results indicate that our main findings of reduced amygdala volume were not affected by effects of intraventricular hemorrhage.

Second, as linked brain regions may develop in concert for their structural features and as such related structural development is altered in human prematurity for selected groups of brain regions as indicated by altered structural covariance^50–52^, we investigated related volumetric features among the left and right amygdalae by a structural covariance approach.

In order to investigate structural covariance across hemispheres, we tested the correlation between left and right whole amygdala volume. The correlation between left and right whole amygdala volume was significant in both the VP/VLBW group (r = 0.865, *p* < 0.001) and the FT controls (r = 0.778, *p* < 0.001), indicating related whole amygdala development across hemispheres. The correlation did not differ significantly between the two groups (*p* = 0.052). However, it approximated statistical significance towards stronger correlation in the VP/VLBW group compared to FT controls, possibly suggesting that related whole amygdala development across hemispheres could be affected by prematurity. Further studies are needed to shed more light on structural covariance within the amygdalae. Table S5 presents correlation coefficients and 95% confidence intervals from the partial correlation analyses, and the *p*-value from comparing correlation coefficients between VP/VLBW subjects and FT controls.

Third, in order to verify that amygdala volume changes were indeed related to premature birth, we investigated whether group differences of amygdala volumes between VP/VLBW subjects and FT controls are specifically related to premature birth using partial correlation analyses with TIV, sex and scanner as covariates. After FDR correction for multiple comparisons, we observed a significant positive correlation between GA and both left (r = 0.367, *p* < 0.001) and right whole amygdala volume (r = 0.317, *p* = 0.002). While there was no significant relationship between BW and left whole amygdala volume (r = 0.106, *p* = 0.304), BW and right whole amygdala volume showed a significant positive correlation (r = 0.220, *p* = 0.031). We found significant negative correlations between duration of ventilation and both left (r = -0.428, *p* < 0.001) and right whole amygdala volumes (r = -0.413, *p* < 0.001). Figure 2b shows the relationship between whole amygdala volumes and variables of premature birth, and Table S6 present correlation coefficients, 95% confidence intervals and *p*-values from the partial correlation analysis between whole amygdala volumes and variables of premature birth.

In summary, our findings support the hypothesis that amygdala volumes are altered in VP/VLBW subjects compared to FT controls and that these volume reductions are specifically related to premature birth.

### Increased social anxiety is not associated with reduced amygdala volumes in premature-born adults

**Figure 3:**
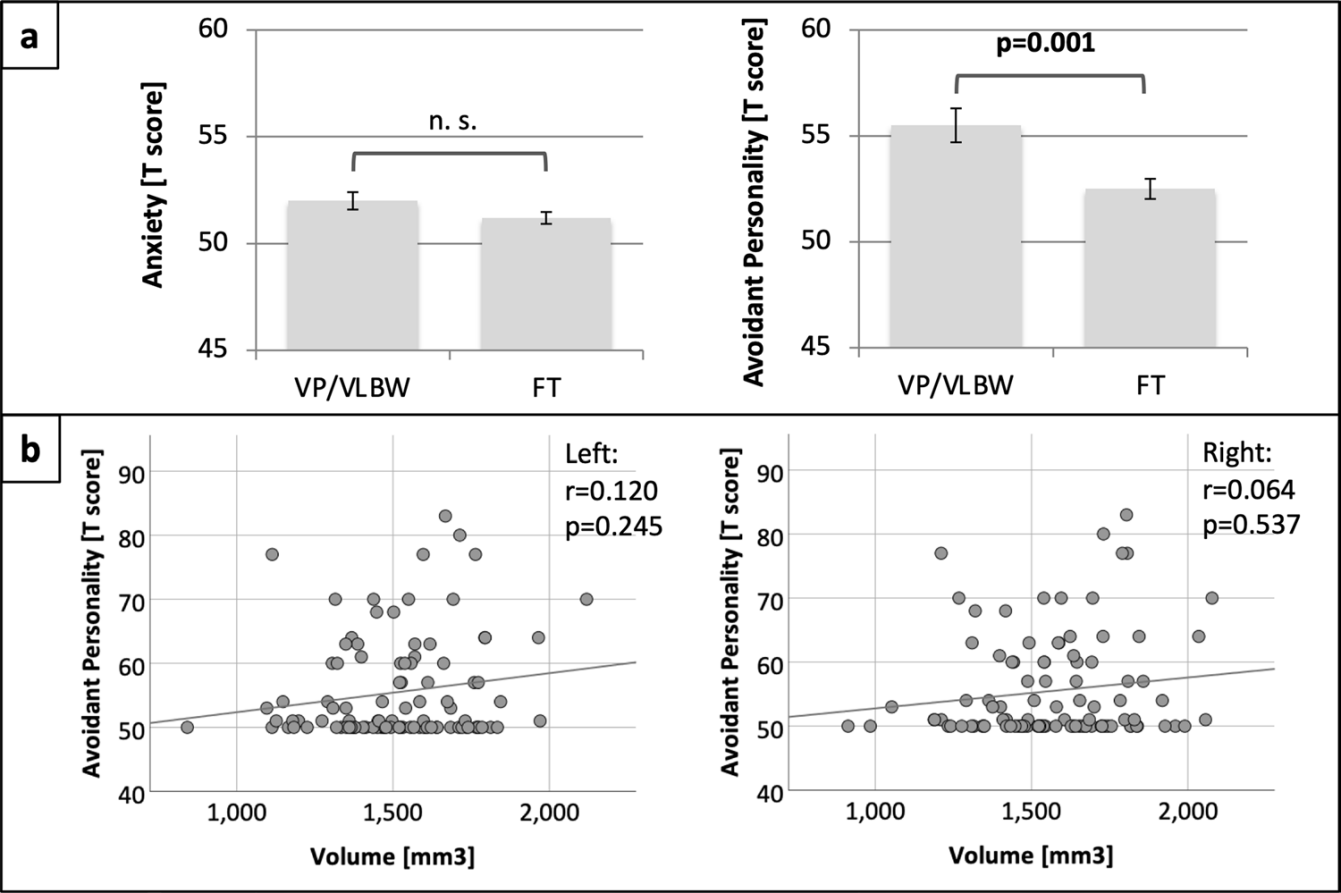
(**A**) Group comparisons between YASR anxiety and avoidant personality scores. Mean T scores are shown as bar charts with SE as error bars. Bold letters indicate statistical significance. (**B**) Relationship between whole amygdala volume and avoidant personality score. The associations between left and right whole amygdala volume and the avoidant personality T score are shown as scatter plots. Whole amygdala volumes (in mm^3^) are plotted on the x-axes. The T score is plotted on the y-axes. Linear regression lines as well as correlation coefficients and *p*-values were added. Bold letters indicate statistical significance after FDR correction using the Benjamini–Hochberg procedure. BW, birth weight; FT, full term; GA, gestational age; L, left; n. s., not significant; R, right; VP/VLWB, very preterm and/or very low birth weight.

To investigate whether premature-born adults had increased anxiety traits, we studied group differences of YASR anxiety and avoidant personality scores by the use of two-samples t-tests. Anxiety scores did not differ significantly between VP/VLBW individuals and FT controls (*p* = 0.097). Avoidant personality scores were significantly higher in VP/VLBW individuals compared to FT controls (*p* = 0.001), indicating increased social anxiety traits in premature-born adults. Figure 3a shows mean T scores as bar charts and Table 1 presents T scores and *p*-values.

In order to study whether reduced amygdala volumes are relevant for increased social anxiety in premature-born adults, we investigated the relationship between amygdala volumes and the YASR avoidant personality score using partial correlation analyses with TIV, sex and scanner as covariates. There was no significant correlation between the T score for avoidant personality and left (r = 0.120, *p* = 0.245) or right whole amygdala volume (r = 0.064, *p* = 0.537). Figure 3b shows the relationship between whole amygdala volume and avoidant personality score and Table S7 presents correlation coefficients, 95% confidence intervals and *p*-values.

These results indicate that VP/VLBW individuals have increased avoidant personality traits, which reflect increased social anxiety; but it seems unlikely that altered amygdala volumes contribute to this trait.

## Discussion

Based on structural MRI, we demonstrated that whole amygdala volumes are decreased in VP/VLBW subjects compared to FT controls at 26 years of age. Furthermore, while VP/VLBW individuals had higher avoidant personality T scores, i.e., they had stronger social anxiety trait, there was no significant association between altered amygdala volumes and social anxiety. To the best of our knowledge, these results demonstrate for the first time decreased amygdala volume in adults of premature birth. Data suggest that prematurity has lasting effects on amygdala volume.

### Reduced amygdala volumes in premature-born adults

We found significantly reduced whole amygdala volumes in VP/VLBW subjects compared to FT controls. Findings in neonates and children after premature birth are mostly in line with our results. At term-equivalent age, reduced amygdala volumes compared to FT controls were reported by Cismaru et al.^37^. While late preterm children showed no significant difference in amygdala volume^53^, children born between 26 and 33 weeks of GA had smaller amygdala volume compared to term controls at school age^36^.

Furthermore, coordinated developmental processes may be reflected by structural covariance^50–52,54^. More specifically, after premature birth, reduced as well as increased covariance has been reported between brain volumes including cortical areas, thalamus and cerebellum^51,52^. We found significant correlation between left and right whole amygdala volume in both the VP/VLBW group and the FT controls, indicating related whole amygdala development. While the correlation did not differ significantly between the two groups, it approximated statistical significance towards stronger correlation in the VP/VLBW group compared to FT controls (*p* = 0.052). This result might suggest that related whole amygdala development across hemispheres could be affected by prematurity. Further studies are needed to shed more light on structural covariance within the amygdalae.

To test whether differences in amygdala volumes between VP/VLBW subjects and FT controls are specifically related to premature birth, we correlated amygdala volumes with variables of premature birth. In general, amygdala volume was consistently related to GA and duration of ventilation, and partly with BW. Our results are in line with findings from other studies at term equivalent age and in children, which found significant correlation between whole amygdala volumes and GA^38,55^. Furthermore, Chau et al.^38^ reported that lower amygdala volume at 8 years was associated with more neonatal invasive procedures and more days of ventilation. Consistent with these findings, our data also showed strong negative correlation of amygdala volume in early adulthood with days of ventilation. This relationship might reflect vulnerability of amygdala to stress exposure induced by premature birth. Scheinost et al.^56^ reporting that also prenatal stress altered amygdala functional connectivity in preterm neonates furthermore supports that stress exposure modulates amygdala development.

In conclusion, our data suggest that amygdala volume is lastingly affected by premature birth.

### Increased social anxiety is not associated with reduced amygdala volumes in premature-born adults

VP/VLBW individuals showed stronger trait social anxiety, as avoidant personality T scores were significantly higher in the VP/VLBW group compared to the FT group. However, there was no significant association between altered amygdala volumes and social anxiety.

These results are in line with Johnson et al.^57^ who found higher scores on avoidant personality scales in extremely preterm adults. Furthermore, they are in line with findings that VP/VLBW are less extraverted and more withdrawn^47,48^. On the other hand, we found no support for the hypothesis that observed reductions in amygdala volumes after premature birth may be associated with anxiety traits. There are several possible reasons why we found no association between increased social anxiety and amygdala volume. First, results about the relevance of amygdala volume for anxiety are heterogeneous. For example, in patients with panic disorder, amygdala volume was significantly smaller compared to controls which was associated with anxiety^58^. Also, in healthy adults an inverse correlation between anxiety measures and volume of the amygdala has been described^46^. Conversely, in children with generalized anxiety disorder, amygdala volume was significantly larger compared to controls^59^. More specifically, enlargement of basolateral amygdala was associated with high childhood anxiety^60^. Likewise, adults with generalized anxiety disorder had larger amygdala volumes^61^. Furthermore, a number of studies found no relationships between personality traits and amygdala volume in relatively healthy adults^62,63^. Second, the amygdala is a region in which highly complex integration takes place^64^ and different aspects of amygdala structure and function contribute to social anxiety. For example, altered structural and functional connectivity of the amygdala have been linked to anxiety^60,65–68^. In particular, social impairments in preterm-born adolescents were associated with functional connectivity of the amygdala, supporting a possible relationship between prematurity, social anxiety and the amygdala^49^. Third, there are several systems contributing to anxiety besides the amygdala^69–71^. For example, apart from the relationship with amygdala volume, Spampinato et al.^46^ found an inverse correlation between anxiety measures and volumes of other regions of the limbic system as well as prefrontal cortex in healthy adults. Lastly, while the 95% confidence intervals reported in Table S7 suggest that we can reject the hypothesis that there is a medium to strong correlation between amygdala volume and social anxiety, there is a possibility that there is only weak correlation which we were not able to detect.

In conclusion, further investigations exploring other modalities and other systems as well as their connectivity patterns are necessary to elucidate neural correlates of social anxiety after premature birth.

### Strengths and limitations

One of the strengths of our study is a large sample size (101 VP/VLBW and 108 FT adults) that enhances the generalizability of our findings. We showed that the sample mean of our data is likely to be close to the ‘true’ population mean and that uncertainty in the estimation of amygdala volumes is relatively low as indicated by small standard errors and narrow 95% confidence intervals (see Tables S1, and S2).

Another strength of our study is that VP/VLBW subjects and FT controls had the same mean age of 26 years. Hence, a relevant impact of patient age on amygdala volumes at the time of the MRI scan is excluded.

Third, segmentation quality was improved as both high-resolution T1-weighted and T2-weighted images were used for amygdala segmentation.

One limitation of this study is that structural MRI of this dataset has previously been analyzed (see methods section). We are aware of the proposed problem of dataset decay—analog to open datasets—increasing the risk of false-positives^72^. However, we analyze this large, prospectively collected cohort of premature-born adults and healthy controls to investigate biologically motivated questions and focused hypotheses. With multiple brain systems affected in human prematurity, it is a first step to focus on distinct brain regions, and a second step to then integrate previous findings, hopefully improving the understanding of brain development after premature birth.

Second, brain structure and its relationship with variables of premature birth as well as social anxiety trait is influenced by multiple other individual, social and environmental factors. Therefore, results have to be interpreted with care.

Third, since individuals with more birth complications in the initial Bavarian Longitudinal Study sample were more likely to be excluded in the initial screening for MRI due to exclusion criteria for MRI, the current sample is biased to VP/VLBW adults with less severe neonatal complications. Therefore, the observed differences in amygdala volumes between VP/VLBW subjects and FT controls are conservative estimates of true differences. Nevertheless, the sample with MRI data was still representative of the full cohort in terms of GA and BW. Mean GA and BW were not significantly different in VP/VLBW subjects with MRI data compared to subjects without MRI data (see Table S8). There were few subjects with intraventricular hemorrhage in the neonatal period (see Table S3). To investigate whether removing subjects with intraventricular hemorrhage impacts the results, we repeated general linear model analyses for left and right whole amygdala volumes (see Table S4) between the remaining subjects of the VP/VLBW group and the FT group. These results indicate that our main findings of reduced amygdala volume were not affected by effects of intraventricular hemorrhage.

## Conclusions

Amygdala volume is lastingly reduced after premature birth. Furthermore, premature-born individuals showed stronger trait social anxiety, however, there seems to be no association between altered whole amygdala volumes and social anxiety.

## Methods

### Participants

Our study sample was previously described in^12,73–75^: All subjects were part of the Bavarian Longitudinal Study (BLS), a geographically defined, whole-population sample of neonatal at-risk children and healthy FT controls who were followed from birth, between January 1985 and March 1986, into adulthood^73–75^. 682 infants were born VP (< 32 weeks of gestation) and/or with very low birth weight (VLBW, birth weight < 1500 g). Informed consent from a parent and/or legal guardian was obtained. From the initial 916 FT born infants born at the same obstetric hospitals that were alive at 6 years, 350 were randomly selected as control subjects within the stratification variables of sex and family socioeconomic status in order to be comparable with the VP/VLBW sample. Of these, 411 VP/VLBW individuals and 308 controls were eligible for the 26-year follow-up assessment. 260 from the VP/VLBW group and 229 controls participated in psychological assessments^13^. All subjects were screened for MR-related exclusion criteria including (self-reported): claustrophobia, inability to lie still for > 30 min, unstable medical conditions (e.g. severe asthma), epilepsy, tinnitus, pregnancy, non-removable MRI-incompatible metal implants and a history of severe CNS trauma or disease that would impair further analysis of the data. However, the most frequent reason not to perform the MRI exam was a lack of motivation. Finally, 101 VP/VLBW subjects and 111 FT controls underwent MRI at 26 years of age (see Fig. S1). The MRI examinations took place at two sites: The Department of Neuroradiology, Klinikum rechts der Isar, Technische Universität München, (n = 145) and the Department of Radiology, University Hospital of Bonn (n = 67). The study was carried out in accordance with the Declaration of Helsinki and was approved by the local ethics committee of the Klinikum rechts der Isar, Technische Universität München and the University Hospital Bonn. All study participants gave written informed consent. They received travel expenses and a small payment for participation.

Previous analyses of this dataset using structural MRI include investigations of cortical architecture^7,8,12^, white matter^15,27,28^, subcortical structures^9,10^, and incidental findings on routine brain MRI^76^.

### Birth variables

GA in weeks was estimated from maternal reports on the first day of the last menstrual period and serial ultrasounds during pregnancy. In cases in which the two measures differed by more than 2 weeks, clinical assessment at birth with the Dubowitz method was applied^77^. BW in grams was obtained from obstetric records. Duration of mechanical ventilation in days was computed from daily records by research nurses.

### Variables related to anxiety

To assess behavioral and emotional outcome related to anxiety, we used the German version of the Young Adult Self Report (YASR)^78^. We chose two (Anxiety and Avoidant personality) of six (Depressive, Anxiety, Somatic, Avoidant personality, Attention deficit/hyperactivity problems, and Antisocial personality) included DSM-IV-oriented scales, which are related to anxiety and social anxiety. Avoidant personality disorder and social anxiety disorder share similar symptoms, such as anxiety in social situations and avoidance of these situations up to self-isolation, and diagnostic criteria largely overlap^79^. We used T scores of the scales as outcome measures. A T score in psychometric testing is a standardized measurement with an average score of 50 and a standard deviation of 10.

### MRI data acquisition

MRI data acquisition was previously described in^9,12^: At both sites, Bonn and Munich, MRI data acquisition was performed on Philips Achieva 3T TX systems or Philips Ingenia 3T system using an 8-channel SENSE head coil. Subject distribution among scanners: Bonn Achieva 3T: 5 VP/VLBW, 12 FT, Bonn Ingenia 3T: 33 VP/VLBW, 17 FT, Munich Achieva 3T: 60 VP/VLBW, 65 FT, Munich Ingenia 3T: 3 VP/VLBW, 17 FT. To account for possible confounds by scanner differences, data analyses included scanner dummy-variables as covariates of no interest. Across all scanners, sequence parameters were kept identical. Scanners were checked regularly to provide optimal scanning conditions and MRI physicists at the University Hospital Bonn and Klinikum rechts der Isar regularly scanned imaging phantoms, to ensure within-scanner signal stability over time. Signal-to-noise ratio was not significantly different between scanners (one-way ANOVA with factor ‘scanner-ID’ [Bonn 1, Bonn 2, Munich 1, Munich 2]; F(3,182) = 1.84, *p* = 0.11). A high-resolution T1-weighted 3D-MPRAGE sequence (TI = 1300 ms, TR = 7.7 ms, TE = 3.9 ms, flip angle = 15°, field of view = 256 mm × 256 mm, reconstruction matrix = 256 × 256; reconstructed isotropic voxel size = 1 mm^3^) and a high resolution T2-weighted 3D sequence (TR = 2500 ms, TE = 364 ms, flip angle = 90°; field of view = 512 mm × 512 mm, echo train length = 120, reconstructed isotropic voxel size = 0.5 mm^3^) were acquired. All images were visually inspected for artifacts.

### MRI processing and amygdala segmentation

Images saved as DICOMs were converted to Nifti-format using dcm2nii^80^. MRI data were segmented using the FreeSurfer image analysis suite, version 6.0 (http://surfer.nmr.mgh.harvard.edu/). It includes a tool to produce an automated segmentation of the amygdala^81^. This atlas was created based on Bayesian inference using ultra high-resolution ex-vivo MRI data and evaluated by applying it to in-vivo MRI data of patients with Alzheimer’s Disease and patients with Autism Spectrum disorders as well as healthy controls^81^. Using both high-resolution T1-weighted and T2-weighted images, the function *segmentHA_T2.sh* generates whole amygdala volumes for the left and right hemisphere, respectively. Output segmentations were inspected visually. Amygdala segmentation is shown in Fig. 1. Successful segmentation of the amygdala was available in 101 VP/VLBW subjects and 109 FT subjects (see Fig. S1).

Estimation of TIV was performed with the CAT12 toolbox, version r1364 (http://www.neuro.uni-jena.de/cat/) ^82^ within SPM12 (https://www.fil.ion.ucl.ac.uk/spm/software/spm12/).

### Statistical analysis

To ensure statistical power, we conducted an a priori power analysis using G*Power^83^. A previous study investigating amygdala volumes after preterm birth at school age found smaller amygdala volume compared to term controls observing large effect sizes^36^. However, since this sample is only partly comparable to our sample, we chose a smaller, medium effect size to calculate sample size. Aiming at obtaining statistical power of 90% at 0.05 level of significance. a sample size of 171 would have sufficed to detect statistically significant results. Hence, our sample size is a very conservative estimate.

All statistical analyses were performed using IBM SPSS Version 26 (IBM Corp., Armonk, NY, USA). First, we checked the data for outliers using a method proposed by Hoaglin and Iglewicz^84^. The interquartile range was multiplied by the factor 2.2. One FT subject was excluded from analyses because left whole amygdala volume was an outlier value. Finally, 101 VP/VLBW subjects and 108 FT subjects were included in the analyses (see Fig. S1).

Age was not included as a covariate in our analyses, as VP/VLBW subjects and FT controls had the same mean age of 26 years (*p* = 0.165).

### Group comparisons of amygdala volumes

To test whether amygdala volumes are significantly altered in VP/VLBW individuals compared to FT controls, general linear models were used. We entered left and right whole amygdala volumes as dependent variables, group membership as fixed factor and TIV, sex and scanner as covariates.

There were few subjects with intraventricular hemorrhage in the neonatal period (see Table S3). To investigate whether removing these subjects impacts the results, we repeated general linear model analyses for left and right whole amygdala between the remaining subjects of the VP/VLBW group and the FT group. To test whether amygdala volumes are significantly altered in VP/VLBW individuals without intraventricular hemorrhage compared to FT controls, we entered left and right whole amygdala volumes as dependent variables, group membership as fixed factor and TIV, sex and scanner as covariates.

To study interrelated development among left and right amygdala, we investigated structural covariance. This approach is motivated by the general notion that linked brain regions may develop in concert, including volumetric features, and that such concerted development seems to be affected by prematurity^50–52^. To investigate structural covariance in the amygdala, we tested the correlation between amygdala volumes. We entered left whole amygdala volume and right whole amygdala volume as variables of interest into a two-tailed partial correlation analysis in each group (VP/VLBW and FT) separately. TIV, sex and scanner were entered as covariates.

Furthermore, we investigated difference in structural covariance between VP/VLBW subjects and FT controls. Using Fisher r-to-z transformation, we calculated the z-score and *p*-value to assess the significance of the difference^85^.

To test whether differences in amygdala volumes between VP/VLBW subjects and FT controls are specifically related to premature birth, we correlated amygdala volumes with GA, BW and duration of ventilation as variables of premature birth. Specifically, in the VP/VLBW group, we entered left and right whole amygdala volumes, respectively, and GA, BW and duration of ventilation as variables of interest and TIV, sex and scanner as covariates into a two-tailed partial correlation analysis.

All analyses were FDR corrected for multiple comparisons using the Benjamini–Hochberg procedure^86^. Statistical significance was defined as *p* < 0.05. 95% confidence intervals for partial correlation analyses were obtained using a bootstrap approach (with 5000 repetitions) in SPSS.

### Correlation of amygdala volumes and anxiety

To investigate the relationship between altered amygdala volumes and anxiety, we used the anxiety and avoidant personality subscales of the YASR DSM-IV-oriented scales. First, we compared YASR anxiety and avoidant personality scores between the VP/VLBW group and the FT control group using a two-sample t-test.

Second, in the VP/VLBW group, we entered left and right whole amygdala volumes, respectively, and anxiety scores (i.e., the avoidant personality T scores) as variables of interest and TIV, sex and scanner as covariates into a two-tailed partial correlation analysis. The partial correlation analysis was FDR corrected for multiple comparisons using the Benjamini–Hochberg procedure^86^. Statistical significance was defined as *p* < 0.05. 95% confidence intervals were obtained using a bootstrap approach (with 5000 repetitions) in SPSS.

## Supplementary Information

Supplementary Information

## Data Availability

Patient data used in this study are not publicly available but stored by the principal investigators of the Bavarian Longitudinal Study.

## Abbreviations

BLS: Bavarian longitudinal study
BW: Birth weight
CI: Confidence interval
CNS: Central nerve system
DSM: Diagnostic and statistical manual of mental disorders
FDR: False discovery rate
FT: Full-term
GA: Gestational age
IQ: Intelligence quotient
MPRAGE: Magnetization prepared rapid acquisition gradient echo
MRI: Magnetic resonance imaging
SE: Standard error
SPN: Subplate neuron
TE: Echo time
TI: Inversion time
TIV: Total intracranial volume
TR: Repetition time
VLBW: Very low birth weight
VP: Very preterm
VP/VLBW: Very preterm and/or very low birth weight
YASR: Young adult self report
