# Supplementary material for: Decreased amygdala volume in adults after premature birth
Source: Sci Rep. 2021 Mar 8;11:5403. doi: 10.1038/s41598-021-84906-2 (PMC7970879; doi:10.1038/s41598-021-84906-2)
Supplement: Supplementary file 1 — Supplementary Information [file 41598_2021_84906_MOESM1_ESM.docx]

**- Original Investigation -**

**Title:** Decreased amygdala volume in adults after premature birth

**Authors and Affiliations:**

Benita Schmitz-Koep, MD ^1,2^*; Juliana Zimmermann, MSc ^1,2^; Aurore Menegaux, PhD ^1,2^; Rachel Nuttall, MSc ^1,2^; Josef G. Bäuml, PhD ^1,2^; Sebastian C. Schneider, BSc ^1,2^; Marcel Daamen, Dr. phil. ^3,4^; Henning Boecker, MD ^3^; Claus Zimmer, MD ^1,2^; Dieter Wolke, PhD ^5,6^; Peter Bartmann, MD ^4^; Dennis M. Hedderich, MD, MHBA ^1,2+^; Christian Sorg, MD ^1,2,7+^

^1^Department of Diagnostic and Interventional Neuroradiology, School of Medicine, Technical University of Munich, Ismaninger Str. 22, 81675 Munich, Germany;

^2^TUM-NIC Neuroimaging Center, School of Medicine, Technical University of Munich, Ismaninger Str. 22, 81675 Munich, Germany;

^3^Functional Neuroimaging Group, Department of Diagnostic and Interventional Radiology, University Hospital Bonn, Venusberg-Campus 1, Bonn, Germany;

^4^Department of Neonatology, University Hospital Bonn, Venusberg-Campus 1, Bonn, Germany;

^5^Department of Psychology, University of Warwick, University Road, Coventry, CV4 7AL, United Kingdom;

^6^Warwick Medical School, University of Warwick, University Road, Coventry, CV4 7AL, United Kingdom;

^7^Department of Psychiatry, School of Medicine, Technical University of Munich, Ismaninger Str. 22, 81675 Munich, Germany.

^+^These authors contributed equally to this work.

**Supplementary Figures**

**Figure S1: Participants of the Bavarian Longitudinal Study**

**
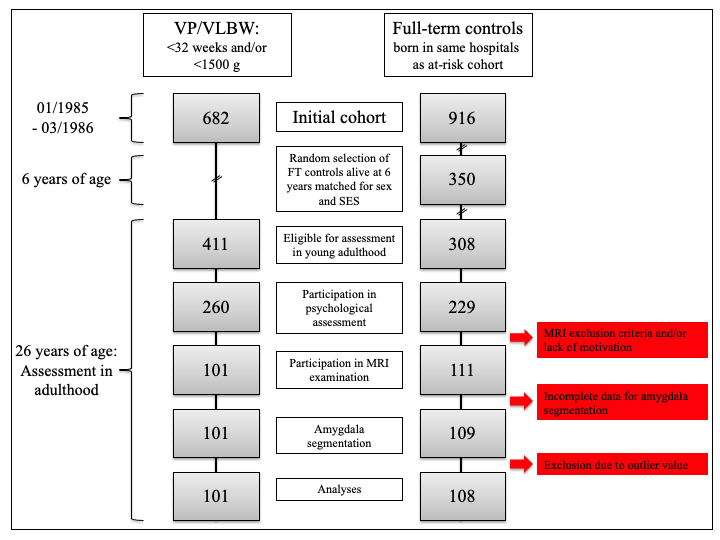
**

Flowchart of the participants of the Bavarian Longitudinal Study.

Abbreviations: MRI, magnetic resonance imaging; SES, socioeconomic status; VP/VLBW, very preterm and/or very low birth weight.

**Supplementary Tables**

**Table S1: Group comparison of whole amygdala volumes**

|  | **VP/VLBW (n = 101)** | | | | **FT (n = 108)** | | | | **p-value** |
| --- | --- | --- | --- | --- | --- | --- | --- | --- | --- |
|  | **mean** | **SE** | **95% CI** | | **mean** | **SE** | **95% CI** | |  |
| Left  Right | 1544.41  1608.57 | 16.46  16.83 | 1511.95  1575.39 | 1576.87  1641.75 | 1660.51  1715.80 | 15.88  16.23 | 1629.21  1683.81 | 1691.82  1747.79 | **<0.001**  **<0.001** |

Estimated marginal means of left and right whole amygdala volume (in mm^3^) with SE (in mm^3^) and 95% CI (in mm^3^) in VP/VLBW subjects and in FT controls. General linear models with group membership as fixed factor and TIV, sex and scanner as covariates. Bold letters indicate statistical significance.

Abbreviations: CI, confidence interval; FT, full term; SE, standard error; VP/VLWB, very preterm and/or very low birth weight.

**Table S2: Raw amygdala volumes**

|  | **VP/VLBW (n = 101)** | | **FT (n = 108)** | |
| --- | --- | --- | --- | --- |
|  | **mean** | **SE** | **mean** | **SE** |
| Left  Right | 1513.24  1564.48 | 21.55  22.91 | 1689.66  1757.03 | 17.32  19.24 |

Raw whole amygdala volumes (in mm^3^) with SE (in mm^3^) in VP/VLBW subjects and in FT controls.

Abbreviations: FT, full term; SE, standard error; VP/VLWB, very preterm and/or very low birth weight.

**Table S3: Intraventricular hemorrhage**

|  | **VP/VLBW (n=101)** | | |
| --- | --- | --- | --- |
|  | **n** | **%** |  |
| **Intraventricular hemorrhage**  **None**  **Stage 1**  **Stage 2**  **Stage 3**  **Stage 4** | 85  5  7  3  1 | 84.2  5.0  6.9  3.0  1.0 |  |

Number of VP/VLBW subjects with intraventricular hemorrhage and its grade assessed with ultrasound examinations in the neonatal period, graded 1-4.

Abbreviations: MRI, magnetic resonance imaging; VP/VLBW, very preterm and/or very low birth weight.

**Table S4: Group comparison of amygdala volumes between VP/VLBW subjects without intraventricular hemorrhage and FT controls**

|  | **VP/VLBW (n = 85)** | | | | **FT (n = 108)** | | | | **p-value** |
| --- | --- | --- | --- | --- | --- | --- | --- | --- | --- |
|  | **mean** | **SE** | **95% CI** | | **mean** | **SE** | **95% CI** | |  |
| Left  Right | 1573.71  1638.41 | 16.98  17.66 | 1511.95  1603.57 | 1540.22  1673.24 | 1661.97  1717.77 | 14.92  15.51 | 1632.54  1687.16 | 1691.39  1748.37 | **<0.001**  **0.001** |

Estimated marginal means of left and right whole amygdala volume (in mm^3^) with SE (in mm^3^) and 95% CI (in mm^3^) in VP/VLBW subjects without intraventricular hemorrhage and in FT controls. General linear models with group membership as fixed factor and TIV, sex and scanner as covariates. Bold letters indicate statistical significance.

Abbreviations: CI, confidence interval; FT, full term; SE, standard error; VP/VLWB, very preterm and/or very low birth weight.

**Table S5:** **Correlation between amygdala volumes**

| **Volume** | **Volume** | **VP/VLBW (n = 101)**  **Correlation coefficient** | **95% CI** | | **FT**  **(n = 108)**  **Correlation coefficient** | **95% CI** | | **z-score** | **p-value** |
| --- | --- | --- | --- | --- | --- | --- | --- | --- | --- |
| Left | Right | 0.865 | 0.792 | 0.915 | 0.778 | 0.673 | 0.852 | 1.941 | 0.052 |

Correlation coefficients and 95% confidence intervals from partial correlation analyses between left and right whole amygdala volumes. TIV, sex and scanner were entered as covariates. Z-score and p-value from comparing correlations.

Abbreviations: FT, full term; VP/VLWB, very preterm and/or very low birth weight.

**Table S6:** **Relationship between amygdala volume and variables of premature birth**

| **Risk factor** | **Volume** | **Correlation coefficient** | **95% CI** | | **p-value** |
| --- | --- | --- | --- | --- | --- |
| GA | Left  Right | 0.367  0.317 | 0.192  0.138 | 0.524  0.472 | **<0.001**  **0.002** |
| BW | Left  Right | 0.106  0.220 | -0.110  0.004 | 0.320  0.428 | 0.304  **0.031** |
| Duration of Ventilation | Left  Right | -0.428  -0.413 | -0.595  0.611 | -0.252  -0.198 | **<0.001**  **<0.001** |

Correlation coefficients, 95% confidence intervals, and p-values from partial correlation analysis between left and right whole amygdala volume and variables of premature birth. TIV, sex and scanner were entered as covariates. Bold letters indicate statistical significance after FDR correction using the Benjamini-Hochberg procedure.

Abbreviations: BW, birth weight; GA, gestational age.

**Table S7: Relationship between amygdala volume and avoidant personality score**

| **YASR score** | **Volume** | **Correlation coefficient** | **95% CI** | | **p-value** |
| --- | --- | --- | --- | --- | --- |
| Avoidant personality | Left  Right | 0.120  0.064 | -0.096  -0.159 | 0.309  0.268 | 0.245  0.537 |

Correlation coefficients, 95% confidence intervals, and p-values from partial correlation analysis between left and right whole amygdala volume and the avoidant personality score. TIV, sex and scanner were entered as covariates. Bold letters indicate statistical significance after FDR correction using the Benjamini-Hochberg procedure.

Abbreviations: CI, confidence interval; YASR, Young Adult Self Report.

**Table S8: Comparison between VP/VLBW subjects with MRI data and without MRI data**

|  | **VP/VLBW with MRI (n=101)** | | **VP/VLBW without MRI (n=159)** | |  |
| --- | --- | --- | --- | --- | --- |
|  | **Mean** | **SD** | **Mean** | **SD** | **p value** |
| **GA (weeks)** | 30.5 | ± 2.1 | 30.6 | ± 2.3 | 0.656 |
| **BW (g)** | 1324 | ± 313 | 1323 | ± 320 | 0.980 |

Statistical comparisons: GA, BW and FS-IQ with two sample t-tests. Statistical significance was defined as p<0.05.

Abbreviations: BW, birth weight; GA, gestational age; INTI, intensity of neonatal treatment index; IQ, intelligence quotient; SD, standard deviation; MRI, magnetic resonance imaging; VP/VLBW, very preterm and/or very low birth weight.

^a^ Data are based on 97 VP/VLBW subjects with MRI data and 120 VP/VLBW subjects without MRI data.
